# Case Report: Ambient Sensor Signals as Digital Biomarkers for Early Signs of Heart Failure Decompensation

**DOI:** 10.3389/fcvm.2021.617682

**Published:** 2021-02-02

**Authors:** Hugo Saner, Narayan Schuetz, Philipp Buluschek, Guillaume Du Pasquier, Giuseppe Ribaudo, Prabitha Urwyler, Tobias Nef

**Affiliations:** ^1^ARTORG Center for Biomedical Research, Gerontotechnology & Rehabilitation Group, University of Bern, Bern, Switzerland; ^2^University Clinic for Cardiology, University Hospital, Inselspital Bern, Bern, Switzerland; ^3^DomoSafety S.A., Lausanne, Switzerland; ^4^Gruppenpraxis Neuhard, Olten, Switzerland; ^5^Neurorehabilitation Unit, Department of Neurology, University Hospital, Inselspital Bern, Bern, Switzerland

**Keywords:** digital biomarker, heart failure, heart failure decompensation, ambient sensor system, home monitoring

## Abstract

Home monitoring systems are increasingly used to monitor seniors in their apartments for detection of emergency situations. More recently, multimodal ambient sensor systems are also used to monitor digital biomarkers to detect clinically relevant health problems over longer time periods. Clinical signs of HF decompensation including increase of heart rate and respiration rate, decreased physical activity, reduced gait speed, increasing toilet use at night and deterioration of sleep quality have a great potential to be detected by non-intrusive contactless ambient sensor systems and negative changes of these parameters may be used to prevent further deterioration and hospitalization for HF decompensation. This is to our knowledge the first report about the potential of an affordable, contactless, and unobtrusive ambient sensor system for the detection of early signs of HF decompensation based on data with prospective data acquisition and retrospective correlation of the data with clinical events in a 91 year old senior with a serious heart problem over 1 year. The ambient sensor system detected an increase of respiration rate, heart rate, toilet use at night, toss, and turns in bed and a decrease of physical activity weeks before the decompensation. In view of the rapidly increasing prevalence of HF and the related costs for the health care systems and the societies, the real potential of our approach should be evaluated in larger populations of HF patients.

## Introduction

Heart Failure (HF) is a serious health condition affecting at least 26 million people worldwide and is increasing in prevalence. HF expenditures are considerable and will increase dramatically with an aging population. Despite significant advances in therapy and prevention, morbidity and mortality are still high and quality of life is often poor (1).

Exacerbation of chronic heart failure (CHF) with the necessity for hospitalization has a significant impact on hospital ressources. Therefore, a major goal of HF therapy is early recognition of signs and symptoms of HF decompensation to allow timely intervention and with that to prevent hospitalizations and increased morbidity.

Multiple sensor systems are used to monitor physiological parameters, activities of daily living and behavior. Based on the sensors reading, digital biomarkers can be extracted and can be used as indicators for health and disease. Signal acquisition can be done either by object sensors, wearable sensors, or contact-free sensors. Contact-free sensors include cameras, pressure sensors, non-contact capacitively coupled ECG (cECG), radar sensors, and passive infrared (PIR) motion sensors (2).

Sensor-based symptoms' assessment can be used in the hospital and in the homes of patients at risk for cardiovascular diseases to monitor physiological parameters (e.g., heartbeat, respiratory rate) and the current activities of daily living (e.g., eating, sleeping). Based on the sensor's reading, digital biomarkers for common cardiovascular diseases can be extracted. Digital biomarkers are defined as an “*objective, quantifiable physiological and behavioral data that are collected and measured by means of digital devices*” (3) to get objective real-time information about the patient's state. Digital biomarkers can be used to drive patient-specific interventions, as endpoints for clinical studies, or as information for formal and informal caregivers to help them to optimize patient care.

Acquisition of digital biomarkers for remote patient monitoring (RPM) can aid in the detection of HF deterioration. Several strategies for RPM have been developed. In most studies, technology embedded in implantable devices have been used for this purpose. However, based on available evidence, routine use of external RPM devices is not recommended (4). Non-hemodynamic monitoring includes interventions ranging from telephone calls only, weight monitoring to complex multi-variable telemonitoring strategies making it difficult to conclude which component drives the effect (5).

Ambient sensor systems are increasingly used to monitor seniors in their apartment not only for safety reasons but more recently also to monitor physiological parameters, activities of daily living, mobility and cognitive impairment (6, 7). One example is the use ballistocardiography for classification of decompensated HF. Aydemir demonstrated that high quality signals can by collected in a home environment by using this technology and that the data can be used to detect the clinical state of the HF patient (8). Despins et al. have analyzed heart rate and respiration obtained from a bed sensor and analyzed the sensor signals for signs of early detection of HF (9), and most recently the use of continuous wearable monitoring analytics to predict heart failure hospitalization has been reported (10). Wearable devices are very helpful for long-term continuous monitoring in the patient's home. However, wearable sensors are difficult to use in some patient populations because cooperation of the patient is needed. Patients need to wear the sensor during the day and during the night (if sleep monitoring is desired) and they need to recharge the wearable sensor. This is feasible in patient with good cognitive abilities, but very difficult in patients with cognitive impairment (e.g., Alzheimer's disease) where the acceptance and feasibility for using wearable sensors is lower (11). Ambient contact-free sensors have the potential to overcome these problems.

Clinical signs of heart failure decompensation include shortness of breath, rapid or irregular heartbeat, fatigue and weakness, reduced ability to exercise, swelling (edema) of legs, ankles, and feet and increased toilet visits at night (nycturia). These signs—with the exception of edema—could be detected by ambient sensor systems, and associated signals may be used as digital biomarkers for heart failure decompensation (6).

The possibility of detecting HF decompensation by means of a pervasive computing system has been mentioned in the literature without reporting a detailed approach (12). This is to our knowledge the first medically documented report about early signs of HF decompensation based on an affordable, contactless, and unobtrusive ambient sensor system for home monitoring.

## Methods

Data from the presented case has been collected as part of a home monitoring study in Switzerland (StrongAge project), where 24 old- and oldest-old, community-dwelling adults were monitored with a combined ambient-sensor and pervasive computing system in their apartments for 1 year. The aim of the study was to evaluate the potential of modern sensor technology to get timely information about emergencies such as falls and also to evaluate the possibility to get preventive information with regards to health status in the setting of an unobtrusive home monitoring system (6, 13). The research protocol has been approved by the Ethics Committee Kanton Bern CH. The patient gave written informed consent for the study and for the publication of the results.

### Ambient Sensors

Physical activity in the apartment and toilet visits were quantified using a commercially available passive infrared (PIR) motion sensing system (DomoSafety S.A., Switzerland). This system included five PIR motion sensor units and two magnetic door sensors that communicate wireless with a base unit. The motion sensors measure motion in equipped rooms once every 2 s (0.5 Hz). All sensors communicate with the base unit through the Zig Bee protocol. The base unit collects the data and send it to the cloud in real-time using the Global System for Mobile Communications (GSM) network. The subject's kitchen, living room, entrance, bedroom, and bathroom were equipped with one PIR sensor based on the room size of the patients apartment. Motion signals have been collected room by room and combined as total activity in the apartment. Normalized daily PIR sensor activity refers to the time the PIR sensors were registering activity in home, normalized by the total time spent at home for a given day. It is thus corrected for the influence of time spent out of home. Transition time has been calculated as time needed to move from one sensor to another as equivalent for gait speed. A bathroom visit was identified as such, if in a 30 min window, at least one sensor firing in the bathroom was recorded. Night-time was defined as the period from 8 p.m. to 6 a.m. (local time) based on the daily activity habits of this particular patient. The validity of the pervasive computing based continuous physical activity assessment has been previously reported (13). Data processing as well as visualizations were done using the Python programming language version 3.6 (Python Software Foundation).

Heart rate, respiration rate as well as toss-and-turn counts were recorded in bed with the commercially available EMFIT QS device (Emfit Ltd., Finland) which is a contact-free piezoelectric sensor placed under the subject's mattress. These sensors use thin quasi-piezoelectric films that measure even light pressure differences as produced by the beating heart. The EMFIT QS sensor has been fixed under the participant's mattress, in proximity to the chest. The device requires no further manual intervention. Data is transmitted to the cloud in real-time through local WiFi and, subsequently, the GSM network. The device extracts a variety of vital signs, including heart rate, respiration rate, heart rate variability, movements in bed, sleep duration, and sleep onset delay. Recent results suggest that this sensor can accurately measure heart rate and respiration rate (14).

## Correlation of Sensor Data With Ground Truth

Sensor data have been collected continuously and prospectively over 1 year. Ground truth has been evaluated by weekly visits of nursing students and included questions related to the general health status including physical, psychological, and social aspects, activities of daily living, and specific health problems over this time period. Ground truth has been correlated with sensor data retrospectively after 1 year. It is of note that the nursing students did not report any signs of heart failure for this particular patient.

### Medical History

This is a case of a 91 years old man, a former executive with frequent traveling around the world. He is living alone in a two-room apartment in a senior's residence.

The senior had a history of 1-vessel coronary heart disease with subacute anterolateral myocardial infarction, a left ventricular ejection fraction of 38% and one short episode of non-sustained ventricular tachycardia 7 years prior to the first index event of heart failure decompensation. Further diagnoses included arterial hypertension, diabetes, kidney failure (GDIGO stadium G2-G3a), vitamin D deficiency, and adenocarcinoma of the prostate.

The senior did not realize any deterioration of his health status. Due to reduced mobility and feeling asymptomatic, he had very rare visits to the family doctor. On January 30th 2018, neighbors of the senior realized that the health situation was deteriorating and sent the senior to his family doctor for an ambulatory visit. The doctor found clinical signs of biventricular decompensation including pulmonary congestion and peripheral edema. Blood pressure was 120/70 mm Hg and heart rate was 62 bpm under betablocker therapy. The senior was referred to the local hospital for further evaluation and treatment. At the time of hospitalization, he was under stable medication with Aspirin 100 mg/d, Irbesartan 300 mg/d, Torasemid 10 mg/d, and Carvedilol 25 mg/d. BNP was 3,361 ng/l at this time. At the hospital, it has been decided to proceed with a conservative treatment including intensified diuretic therapy with addition of an aldosterone antagonist and a reduction of the betablocker dosage, and a few days later a hospital based geriatric rehabilitation program with physiotherapy, training sessions, optimization of the medication, and interventions to facilitate the return to the live previous to the event has been started. Two weeks later, the senior returned to his home in a compensated status resuming his previous activities.

### Early Signs of Heart Failure Decompensation by the Ambient Sensor System

The sensor system started to indicate clinical signs of heart failure decompensation more than 3 month prior to the hospitalization. Signals from the bed sensor indicated increasing respiration rate, increasing heart rate at night (damped under betablocker therapy), and an increasing number of toss-and-turns in bed. Ambient PIR sensors indicated reduced physical activity during the day, less time spent out of the apartment and increased bathroom visits at night (nycturia). Additional findings include an increase of the room transition time by >25% over 4 month prior to the hospitalization and a decrease of the refrigerator openings from in average 6–7 openings 4 month prior to the hospitalization to 0–1 openings per day during the days before hospitalization.

**Figure 1:**
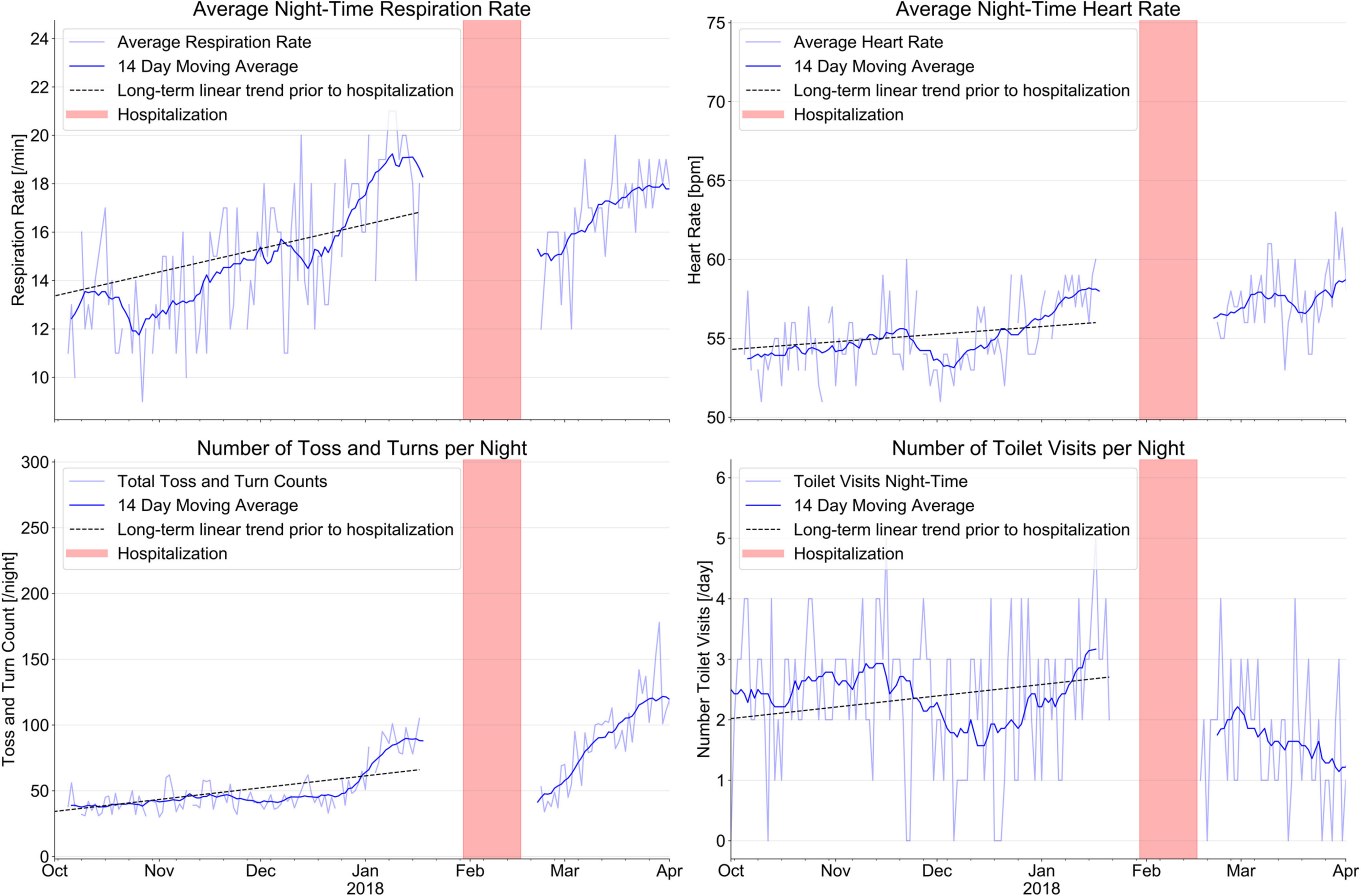
Evolution of sensor signals from a combined ambient passive infrared motion sensor system to monitor the number of toilet visits at night with an EMFIT QS bed sensor device. The combination of signals from the various sensors before hospitalization indicate signs of increasing heart failure decompensation long before the index event on January 30^th^ 2018 (red bars indicate hospitalization). After hospital discharge, sensor signals indicate improvement of all parameters.

Figure 1 shows our results with regard to respiration rate and heart rate at night, bathroom visits at night and toss- and-turns in bed. Mean respiration rate/minute increased from 12.6 ± 2.0/min. three month prior to the hospitalization to 18.4 ± 1.6/min. at the time of hospitalization; mean heart rate increased from 54.0 ± 1.8/min to 57.3 ± 1.6/min under stable betablocker dosage; toss-and-turns in bed increased from 39.2 ± 6 to 81.6 ± 15 and toilet visits at night increased from 2.4 ± 1.0 to 2.7 ± 1.0. Except for toilet use at night, all changes are clinically important.

**Figure 2:**
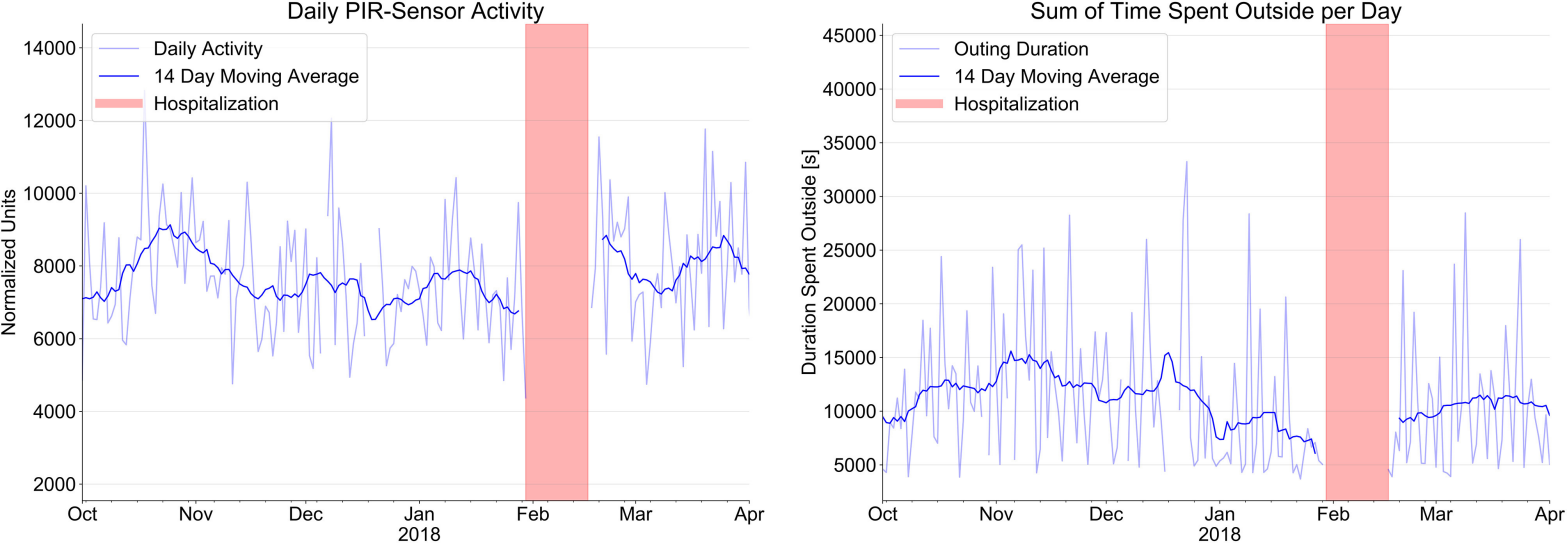
Evolution of sensor signals from passive infrared PIR motion sensor system (normalized units) to indicate daily activity and time spent outside the apartment over a period of 4 month prior to hospitalization (red bars) and after hospital discharge.

Figure 2 shows the number of activity signals and the total time of outings per day over the same time period. We found a steady decline of total physical activity in the apartment over this period of time with a decline of the daily PIR-Sensor activity (normalized units) from 8,113 ± 1,663 to 7,343 ± 1,387 and a marked reduction of the sum of time spent outside per day (min) from 180.6 ± 81.8 min to 132.3 ± 94.1 min.

After hospital discharge and return to home, respiration rate, toss-and-turn in bed, and toilet visits at night decreased, whereas heart rate increased slightly under reduced betablocker therapy. Daily PIR-sensor activity and sum of time spent outside increased also considerably.

## Discussion

This is to our knowledge the first report about the potential of an affordable, contactless, and unobtrusive ambient sensor system for the detection of early signs of HF decompensation based on prospective data acquisition and retrospective correlation of the data with clinical events in a 91 year old senior with a serious heart problem over 1 year. The ambient sensor system detected an increase of respiration rate, heart rate, bathroom visits at night, toss, and turns in bed and a decrease of physical activity weeks before the decompensation.

Our results indicate a great potential of home monitoring signals provided by a non-obtrusive ambient sensor system for the detection of early signs of heart failure decompensation. Clinical signs such as shortness of breath with increasing respiration rate at night, changes in heart rate, nycturia with increasing toilet visits at night, toss-and-turns in bed, and fatigue with decreased physical activity can easily be recorded by unobtrusive ambient sensor systems, and health information can be generated by the combination of different signals. Information from sensors may be used as individual digital biomarkers but may indicate different health problems. This has to be taken into account when programming the system: increased respiration rate for instance may also indicate a pulmonary problem, increased heart rate by itself is unspecific, increased toilet use at night may also indicate urinary infection or another urological problem. Decrease of physical activity over time is also an unspecific symptom. However, the combination of such signs and their changes over time may allow to identify heart failure decompensation. Sleep disturbances with increased toss-and -turns in bed and decreased time of home exits are further signs of a serious health problem, which may be additionally be entered into the diagnostic scheme of the pervasive computing system. Combinations of signs may not always allow an accurate diagnosis such as heart failure decompensation but still indicate a serious health problem with a distinct differential diagnosis. Such information can be used for timely information of health care professionals taking care of such a patient. Early alerts for health professionals have a great potential to allow early intervention and by this to reduce or prevent hospitalizations. As the number of hospitalizations for heart failure decompensation in elderly people is high and increasing around the world, avoiding hospitalization by timely intervention has a great potential not only to improve quality of life for the patient but also to save costs for the health care system.

The manuscript has several limitations to be addressed. The major limitation is the fact that it is a single case report and that findings can only be seen as a description of a promising concept of how signals from an ambient sensor system could be used and combined in a way which may allow early diagnosis of heart failure decompensation. The problem that we are not able to present “normal values” with standard deviations for the data is another inherent problem of single case reports. A further limitation is the fact that although sensor data and ground truth have been collected prospectively, the correlation of the data with the clinical situation was performed retrospectively. In addition, a combination of individual digital biomarkers as proposed may indicate a health problem which can be different from heart failure decompensation. However, even if it indicates a different cardiovascular or pulmonary problem, there is a strong indication that the health situation of the patient is serious.

## Conclusions

Ambient sensor systems are increasingly used to monitor seniors in their apartment not only for safety reasons but more recently to detect patterns indicating serious mental or physical problems. Such sensor systems are unobtrusive and allow seniors to keep privacy and independence. Our findings indicate a great potential of contactless pervasive computing systems for detection of early signs of serious health problems such as heart failure decompensation. Further studies with a prospective design are necessary to evaluate the full range of the clinical potential of these findings.

## Data Availability Statement

The original contributions presented in the study are included in the article/supplementary material, further inquiries can be directed to the corresponding author/s.

## Ethics Statement

The studies involving human participants were reviewed and approved by Ethical Committee of the Canton Bern, Bern CH. The patients/participants provided their written informed consent to participate in this study.

## Author Contributions

HS, NS, and PB have been involved in data analysis. HS, NS, and PU have been involved in preparation of the manuscript. All authors contributed to the study design, conception, contributed to data collection, and management. All authors have been involved in data interpretation, reviewed, and approved the manuscript.

## Conflict of Interest

PB and GD are working at Domosafety S.A., one of the manufacturers of the used sensor system. The remaining authors declare that the research was conducted in the absence of any commercial or financial relationships that could be construed as a potential conflict of interest.
